# Exploring burnout and uncertainty in healthcare professionals: a path analysis within the context of rare diseases

**DOI:** 10.3389/fpubh.2025.1417771

**Published:** 2025-02-27

**Authors:** David Zybarth, Laura Inhestern, Corinna Bergelt

**Affiliations:** ^1^Department of Medical Psychology, University Medical Center Hamburg-Eppendorf, Hamburg, Germany; ^2^Department of Medical Psychology, University Medicine Greifswald, Greifswald, Germany

**Keywords:** uncertainty, burnout—professional, psychology, healthcare professional, path analysis, rare disease

## Abstract

Burnout among physicians has gained increasing attention in recent years. This issue arises not only from stressful working conditions and individual factors but also from the correlation between burnout and physicians’ tolerance of uncertainty. This association could be particularly important in the context of rare diseases, which inherently present greater uncertainty. To date, no studies have explored this topic. Our exploratory study aimed to investigate the associations between uncertainty and burnout scores among physicians while considering secondary factors associated with rare diseases and COVID-related stress. Although not the primary focus, we included COVID-related stress due to its impact during the ongoing pandemic. We conducted an online survey using the Physicians’ Reaction to Uncertainty Scale (PRU) and the Oldenburg Burnout Inventory (OLBI). Experience with rare diseases was quantified by assessing the weekly working hours devoted to patients with such conditions. We conducted a path analysis, initially using a fully recursive model and subsequently eliminating non-significant paths. 128 physicians (*n* = 73 female) participated in the survey, with 31% of them displaying significant burnout scores. Notably, significant associations were found between the PRU subscale anxiety and both dimensions of burnout, as well as between the PRU subscale disclosure to patients and the burnout dimension of exhaustion. COVID-related stress was also significantly associated with exhaustion, while experience with rare diseases was significantly associated with disengagement. No correlation was observed between experience with rare diseases and uncertainty scores. The model demonstrated an excellent fit (RMSEA = 0.055). Our results show that physician burnout is a pressing issue and confirm the association between anxiety due to uncertainty and increased burnout scores.

## 1 Introduction

Being passionate about one’s job offers numerous benefits, particularly for healthcare professionals (HCPs, used synonymously with physicians in this article). Passionate HCPs typically show better mental and physical health and achieve higher patient satisfaction (1). Conversely, chronic poses significant risks, potentially causing mental, physical, and behavioral harm to both the individual and their organization (2–4). Burnout syndrome, a critical issue in this context, is characterized by two bipolar core dimensions: exhaustion vs. vigor and identification vs. distancing (5). According to Maslach et al. (6), there is a third dimension: personal accomplishment. However, empirical evidence suggests that personal accomplishment is more likely an antecedent or a consequence of burnout (7). Apart from the number of dimensions, there are different definitions of burnout in the current literature. Some authors use a bidimensional approach, assuming that scores in both core dimensions have to be high [e.g., (8, 9)]. Others use a unidimensional approach, implicating that a high score in one dimension is sufficient [e.g., (10, 11)]. Using the bidimensional definition, prevalence rates for HCPs range between 8 and 29%, with a pooled prevalence estimate of 20%. Considering the unidimensional approach, the pooled estimate doubles (43%) and ranges between 15 and 72% (12). We follow the bidimensional approach assessed with the Oldenburg Burnout Inventory (OLBI; 57). Mostly related issues such as high demands, a lack of recovery activities, and a lack of control are seen as causal for the development of burnout syndrome (13, 14). Additionally, personal variables such as higher levels of neuroticism and maladaptive individual coping strategies (e.g., denial, disengagement, and self-blame) modulate the development of burnout (4, 15, 16). In their review, Patel et al. (17) also identified several organizational factors like negative leadership behaviors and insufficient rewards. For HCPs, high burnout levels are often driven by the intensity and frequency of patient demands (18). The consequences of burnout might be serious. They range from substance use to suicidal ideation and higher probabilities for significant medical errors (17, 19). Therefore, effective coping strategies are essential for HCPs.

Lazarus and Folkman (58) identified three general coping strategies for stress: emotion-focused, problem-focused, and cognitive. If confronted with high job-related demands, several theories suggest that distancing serves as an emotion-focused strategy for coping with exhaustion (4). Following this approach, burnout results from a dysfunctional coping strategy that unfolds stepwise: exhaustion, as a core dimension of burnout, emerges first, followed by distancing as a subsequent consequence (59).

Depending on the individual’s uncertainty (in) tolerance, uncertainties are an additional stressor for HCPs. Uncertainty is defined as “metacognitive awareness of ignorance” and is omnipresent in medicine (20). Uncertainty tolerance refers to an individual’s psychological response to uncertainties, which can be positive or negative. A tendency to interpret uncertainties negatively indicates a lower tolerance for uncertainty (21). This lower tolerance is associated with several behavioral outcomes, such as a reluctance to involve patients in decision-making or to disclose uncertainties (22). Additionally, studies show associations between lower uncertainty tolerance, reduced job satisfaction, and higher burnout rates (23). Some research indicates that medical specialty may influence uncertainty scores, with high rates of less differentiated illnesses correlating with lower uncertainty tolerance (24). An established instrument to measure the uncertainty tolerance of HCPs is the Physicians’ Reaction to Uncertainty Scale [PRU; (25)], which assesses uncertainty through four subscales.

A field of medicine that is inherently associated with high levels of uncertainty is rare diseases. In the European Union, a disease is defined as rare when it affects less than 5 in 10,000 people (26). Due to the low numbers of individuals who are affected by a certain disease, research and knowledge are limited. Consequently, HCPs constantly have to deal with ambiguous situations, enduring a variety of uncertainties like unclear treatment recommendations, prognoses, and explanations for symptoms (27). It remains unclear if this extensive confrontation with uncertainty leads to psychosocial consequences for physicians. Individual studies suggest that working in uncertainty-heavy medical contexts, such as genetic counseling, triggers stress in HCPs (28). Anyway, studies that directly examine the psychosocial impacts of uncertainty in the context of rare diseases are currently lacking (29).

Our study aimed to investigate the associations between uncertainty and burnout scores of HCPs using a path analytical approach. We defined uncertainties as stressors and hypothesized an association between the PRU subscales and both burnout dimensions. This hypothesis aligns with the existing literature (23, 30). To our knowledge, the impact of working with people with rare diseases on the experience of uncertainty and burnout has not yet been investigated. Consequently, we decided to conduct an exploratory assessment of potential correlations between these factors within the path analysis. Additionally, we assessed the extra work stress due to COVID-19, as the survey was conducted during the pandemic. Although it was not our primary focus, considering the added burden of the pandemic on HCPs appeared necessary, as a variety of studies suggest the effects of the pandemic situation on burnout scores of HCPs regardless of their medical specialty (31–33).

## 2 Methods

### 2.1 Study design and recruitment

To explore the relationship between uncertainty, burnout, and experience with rare diseases, we developed a brief online survey for HCPs, regardless of their specialty or experience with rare diseases. No further in- or exclusion criteria were set. Ethical approval for the survey was gained from the Local Psychosocial Ethics Committee of the Center for Psychosocial Medicine at Medical Center Hamburg-Eppendorf (LPEK-0372). All participants actively approved consent to the survey prior to participation.

The cross-sectional survey was designed as a 10-min questionnaire and was conducted between January and December 2022. It was administered by LimeSurvey (version 2.62.2 + 170,203). To ensure anonymity, HCPs were not contacted directly by the study team but via different institutions. We contacted the press offices of the Medical Chambers in each German federal state and the following professional societies: Hartmannbund – Verband der Ärztinnen und Ärzte Deutschlands e. V., Marburger Bund, Deutscher Hausärzteverband e.V., Berufsverband der Kinder- und Jugendärzt*innen e.V., Deutsche Gesellschaft für Kinder- und Jugendmedizin e.V. We requested these organizations to send the description of our survey, including a link, to their members through newsletters and other publications. Additionally, we asked for our survey to be disseminated through newsletters and mailing lists of different organizations (Hamburger Netzwerk für Versorgungsforschung, KEKS e.V., Department of General Practice and Primary Care at Medical Center Hamburg-Eppendorf). We also utilized social media posts featuring a QR code to recruit participants. As participation was voluntary, our analyses were based on a convenience sample.

### 2.2 Burnout

To assess burnout, we used the German version of the Oldenburg Burnout Inventory (OLBI; 57). The instrument is well-validated and is frequently used in HCP samples (60). In its current version, the OLBI consists of 16 items equally measuring the two core dimensions of exhaustion and disengagement on a 4-point Likert scale (1-strongly agree, 4-strongly disagree). Higher scores indicate higher strains on the dimensions. In contrast to the Maslach Burnout Inventory (6), the subscales use positively and negatively worded items equally (61). In line with the literature, we set a cut-off equal to or higher than 2.25 for exhaustion and a cut-off equal to or higher than 2.1 for disengagement (34). For overall burnout, a cut-off equal to or higher than 2.18 was applied (35). Cronbach’s alpha values are greater than 0.7, indicating satisfactory internal consistency (36, 37). Specific values are presented in Table 1.

**Table 1:** Descriptive statistics of stress, burnout, and uncertainty measures in *n* = 128 HCPs.

Variable	*n*	Minimum	Maximum	Mean	Standard deviation	Cronbach’s alpha
COVID-related stress	128	1	10	5.02	2.39	
OLBI exhaustion	128	1.38	3.75	2.46	0.53	0.85
OLBI disengagement	128	1.13	3.25	1.89	0.47	0.75
PRU anxiety	128	4	24	13.37	4.97	0.87
PRU concern	128	4	24	11.75	5.07	0.87
PRU disclosure to patients	128	5	27	12.20	5.59	0.89
PRU disclosure to physicians	128	2	11	3.94	2.23	0.8

OLBI, Oldenburg burnout inventory; PRU, Physicians’ reaction to uncertainty scale.

### 2.3 Uncertainty

The Physicians Reaction to Uncertainty Scale [PRU; (25)] is an instrument widely used to assess HCP’s affective and behavioral responses to uncertainty, which is an indicator of uncertainty tolerance (21, 22). It was translated into German and culturally adapted by Schneider et al. (38) and consists of 15 items assigned to four subscales: anxiety due to uncertainty (PRU anxiety, four items), concern about bad outcomes (PRU concern, four items), reluctance to disclose uncertainty to patients (PRU disclosure patients, five items) and reluctance to disclose mistakes to physicians (PRU disclosure physicians, two items). Participants indicate agreement on a 6-point Likert scale (1-strongly disagree, 6-strongly agree). There is no overall score, but higher scores in subscales indicate higher anxiety, concern, or reluctance to disclose uncertainties to patients or mistakes to physicians. Cronbach’s alpha values are greater than 0.7, indicating satisfactory internal consistency [Table 1; (36, 37)].

### 2.4 Assessments of secondary factors

Sociodemographic characteristics were assessed in categories to ensure anonymity. We asked participants for their gender (male, female, diverse) as well as for their age (<30, 30–40, 41–50, 51–60, >60) and their medical specialty (for an overview of assessed medical specialties see Additional File 1). As an indicator of rare disease experience, participants rated the percentage of their weekly work time spent providing care to people with rare diseases. Participants were provided with the European Union’s definition of a rare disease. We assessed additional work stress due to the pandemic with the single item “How much of a strain is COVID currently putting on your day-to-day work?” using a 10-point Likert scale (1-no strain at all, 10-very heavy strain).

### 2.5 Statistical analyses

Descriptive analyses were conducted using IBM SPSS Statistics 29.0 (IBM Corp, Armonk, New York) to describe the sample. All answers were marked as mandatory. Sociodemographic data were excluded from being mandatory due to data protection regularities. Only fully completed questionnaires were included in the analyses. A total of eight questionnaires had to be excluded from the analyses afterward as essential information (gender or age) was missing. One person had to be excluded from analyses as an outlier, only choosing the first answer option in all items. We used the software IBM SPSS AMOS 27 (IBM Corp, Armonk, New York) to answer the research question. We decided on a path analysis to describe direct and indirect effects and estimate the magnitudes of hypothesized relationships between variables. According to Kline (39), the sample size should be 10 times the number of parameters, resulting in a minimum sample size of 110 participants. To the best of our knowledge, no previous studies examined the associations of interest, so we chose an exploratory approach and started with a full-recursive model. The only exception was the relationship between the uncertainty subscales and the burnout scales already described in the literature (30). Furthermore, we added demographic data and COVID-related stress as they were identified as potential confounders of the variables of interest (40–42). After calculating the first model, we gradually removed the non-significant associations with the greatest *β*-coefficient to focus on relevant paths and identify a parsimonious model. We used a maximum likelihood estimation, and for all analyses, *p*- values of < 0.05 were considered statistically significant. To obtain more robust results, we used the statistical technique of bootstrapping with 500 bootstrap samples. The model fit was assessed by standard indices: CMIN/ DF statistics, root mean square error of approximation (RMSEA), Tucker-Lewis index (TLI), and comparative fit index (CFI). For CMIN/DF, values ≤3 indicate an acceptable fit (39). For RMSEA, Hu and Bentler (43) suggested scores <0.06 as acceptable and scores >0.95 for TLI and CFI.

## 3 Results

### 3.1 Sample characteristics

203 HCPs started the survey by at least clicking on the link provided. Out of these, 128 persons completed the questionnaire, which was adequate for the planned analyses. 57% of participants were female, a majority were younger than 51 years (61%), and 82% had contact with patients with rare diseases. See Table 2 for detailed information.

**Table 2:** Sociodemographic data of *n* = 128 HCPs.

Variable	*n* (%)
Gender	
Male	55 (57)
Female	73 (43)
Diverse	0 (0)
Age group	
30–40	38 (30)
41–50	40 (31)
51–60	29 (23)
> 60	21 (16)
Specialty	
General practitioner/internal medicine	43 (34)
Pediatrician	39 (31)
Other	46 (36)
The weekly proportion of work with people with a rare disease in %	
0	24 (19)
1–30	69 (54)
31–60	15 (12)
61–99	18 (14)
100	2 (2)

### 3.2 Descriptive statistics

Table 1 shows an overview of COVID-related stress, burnout, uncertainty scores, and Cronbach’s alpha values. Applying the cut-off ≥2.25, 65% (*n* = 83) of the participants reported significantly elevated levels of exhaustion. 34% (*n* = 44) exceeded the cut-off in the scale disengagement. Using the bidimensional approach, 31% (*n* = 39) of HCPs could be classified with burnout, as they exceeded cut-offs in both scales. Applying the overall cut-off ≥2.18, 48 = % (*n* = 61) HCPs tested positive for burnout. All Cronbach’s alpha values are greater than 0.7, indicating satisfactory internal consistency (36, 37).

### 3.3 Zero-order correlations

Table 3 presents the zero-order correlations of all included variables. There are small to moderate significant correlations between the PRU subscales, with a strong correlation between the PRU subscales’ anxiety due to uncertainty and concern about bad outcomes. Additionally, a strong, significant correlation exists between both OLBI dimensions. COVID-related stress is significantly associated with both OLBI dimensions and the PRU subscales anxiety due to uncertainty and concern about bad outcomes. The weekly proportion of work with rare diseases shows a small to moderate, significant correlation between the PRU subscale reluctance to disclose uncertainty to physicians and the OLBI dimension disengagement. Gender is small to moderately associated with all PRU subscales, while age shows a significant correlation with the OLBI dimension disengagement.

**Table 3:** Zero-order correlations based on *n* = 128 HCPs.

Variable	1	2	3	4	5	6	7	8	9	10
1. Gender	1									
2. Age	0.31**	1								
3. COVID-related stress	−0.85	0.14	1							
4. Proportion of work with rare diseases	0.00	−0.15	−0.16	1						
5. PRU anxiety	−0.3**	−0.57	0.20*	−0.12	1					
6. PRU concern	−0.39**	0.34	0.23**	−0.13	0.66**	1				
7. PRU disclosure to patients	−0.23**	0.01	0.01	−0.09	0.3**	0.24**	1			
8. PRU disclosure to physicians	0.23**	0.1	0.17	−0.21*	0.24**	0.23**	0.19*	1		
9. OLBI disengagement	−0.14	−0.23**	0.22*	−0.28 **	0.35**	0.28**	0.17	0.19*	1	
10. OLBI exhaustion	−0.11	−0.10	0.32**	−0.13	0.34**	0.30**	0.00	0.17	0.63**	1

OLBI, Oldenburg burnout inventory; PRU, Physicians’ reaction to uncertainty scale. *p < 0.05 (two-tailed); **p < 0.01 (two-tailed).

### 3.4 Path analysis

**Figure 1:**
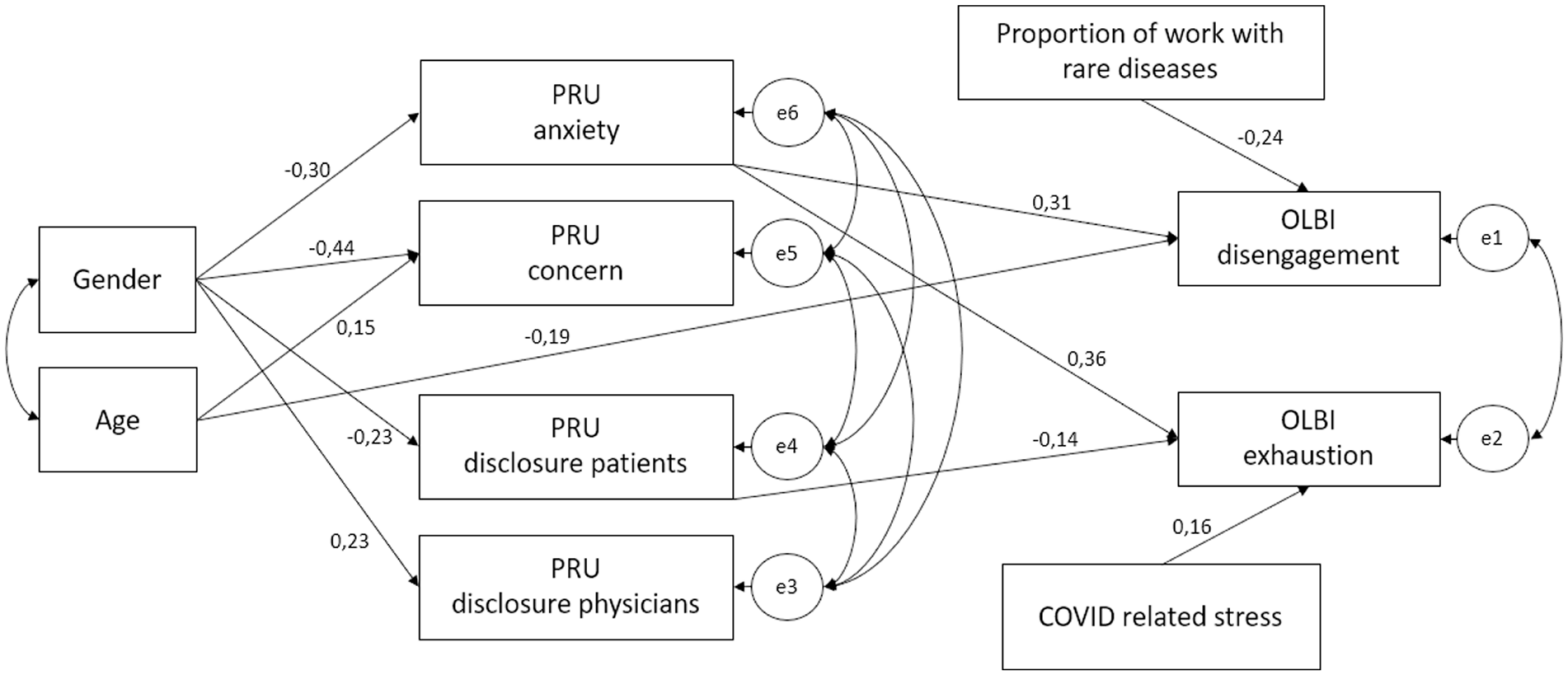
Final path model describing the relationships between exogenous variables (Gender, Age, Proportion of work with rare diseases, and COVID-related stress), uncertainty (subscales of the Physicians’ Reaction to Uncertainty Scale), and burnout (subscales of the Oldenburg Burnout Inventory) in HCPs (*n* = 128); numbers represent standardized regression coefficients (*β*); eX represents error terms.

An exploratory path analysis was conducted to examine the relationships between the subdimensions of uncertainty and burnout, considering the proportion of weekly exposure to rare diseases, COVID-related stress, age, and gender. The full-recursive model (Additional File 2) failed to show an acceptable fit (CMIN/DF = 10.396, RMSEA = 0.272, TLI = -0.613, CFI = 0.964). After gradually removing non-significant associations, our final model showed a very good fit to our observed data (CMIN/DF = 1.389, RMSEA = 0.055, TLI = 0.933, CFI = 0.961). Figure 1 shows the model, including the standardized regression weights. Total effects, covariances, upper and lower bootstrap bounds, and *p*-values are summarized in Table 4. The analysis indicates that gender has only an indirect effect on both OLBI dimensions and a direct small to moderate effect on all PRU subscales. The effect of age on the PRU subscale concern about bad outcomes, and the OLBI dimension disengagement is small. Notably, the PRU subscale anxiety due to uncertainty has a moderate, significant effect on both OLBI dimensions, while the PRU subscale reluctance to disclose uncertainty to patients is negatively associated with the OLBI dimension of exhaustion. The weekly proportion of work with patients with rare diseases is negatively correlated with the OLBI dimension disengagement. Furthermore, COVID-related stress is significantly associated with the OLBI dimension exhaustion.

**Table 4:** Overview of significant, standardized total effects and covariances, incl. bootstrap bounds and *p*-valuescorresponding to the final path model; based on n=128 HCPs.

Effect of	Total effect/covariance	Bootstrap lower bound	Bootstrap upper bound	*P*-value
Gender on				
PRU anxiety	−0.297	−0.434	−0.129	0.006
PRU concern	−0.439	−0.562	−0.272	0.007
PRU disclosure patients	−0.227	−0.394	−0.034	0.008
PRU disclosure physicians	0.231	0.063	0.381	0.015
OLBI disengagement	−0.093	−0.168	−0.028	0.005
OLBI exhaustion	−0.073	−0.167	−0.005	0.034
Age on				
PRU concern	0.149	0.019	0.277	0.026
OLBI disengagement	−0.188	−0.309	−0.043	0.009
COVID-related stress on				
OLBI exhaustion	0.162	0.022	0.295	0.038
Proportion of work with rare diseases on				
OLBI disengagement	−0.237	−0.347	−0.123	0.006
PRU anxiety on				
OLBI disengagement	0.312	0.143	0.453	0.005
OLBI exhaustion	0.355	0.180	0.486	0.006
PRU disclosure patients on				
OLBI exhaustion	−0.144	−0.296	−0.033	0.019

OLBI, Oldenburg burnout inventory; PRU, Physicians’ reaction to uncertainty scale. Bootstrap bounds and *p*-values corresponding to the final path model, based on *n* = 128 HCPs.

## 4 Discussion

This study examined the relationship between uncertainty and burnout, considering possible influences of the weekly proportion spent with people with rare diseases and COVID-related stress. To our knowledge, this is the first study to examine these associations in more detail by analyzing relationships between the dimensions of the constructs.

### 4.1 Burnout

In our sample, about 2/3 of HCPs exceeded the cut-off for exhaustion and about 1/3 for disengagement in the Oldenburg Burnout Inventory (OLBI). Using the bidimensional approach, 31% of the participants are affected by burnout. These results are striking but in line with other studies that use bidimensional definitions (44, 45). While the sample recruited by O’Kelly et al. (44) only consisted of urologists, Sulaiman et al. (45) recruited HCPs irrespective of their medical specialty. Our findings indicate that burnout is a risk that HCPs of all medical specialties have to face. A comprehensive review by Ryan et al. (46) outlines the consequences. The authors identified a robust association between burnout and depression or between anxiety and inconsistent findings, considering the associations between burnout and suicidal ideation or substance abuse.

Besides these serious consequences for the HCPs, burnout will likely affect patient care. It is associated with decreased empathy, poorer quality of care, and increased risk of major medical errors and adverse patient safety incidents (47). Following the stepwise development of burnout with exhaustion leading to disengagement, the future proportion of affected HCPs is likely to rise in our sample. Using the overall cut-off, the rate of HCPs affected by burnout rises to 48% but remains much lower than those of comparable studies performed during the COVID-19 pandemic. Sheehan et al. (35) identified 76% of physicians exceeding the predefined cut-off. The large difference could be attributed to the different times the surveys were conducted. Sheehan et al. (35) performed their survey in an early phase of the pandemic, which was associated with higher burnout prevalence rates than in later phases (48). While COVID-related stress was significantly correlated with the two PRU subscales, anxiety due to uncertainty and concern about bad outcomes in zero-order correlations, the association appears to be modulated by the OLBI dimension exhaustion in the path analysis conducted within our sample.

Additionally, COVID-related stress was only linked to the OLBI scale exhaustion rather than to disengagement. This finding is in line with the idea that exhaustion develops prior disengagement in the progression of burnout (59). Therefore, it highlights the need for support initiatives to prevent an increase in burnout severity among HCPs.

### 4.2 Associations between uncertainty and burnout

As shown in other studies, there are significant associations between uncertainty, measured with the Physicians’ Reaction to Uncertainty Scale (PRU), and burnout scores of HCPs. While the zero-order correlations indicate a broad correlation of the PRU subscales with both dimensions of burnout, the path model, which considers the subscales’ shared variance, limits the associations to the subscales’ anxiety due to uncertainty and reluctance to disclose uncertainty to patients. The first finding aligns with a study conducted by Cooke et al. (30), who also found a significant association between the PRU subscale anxiety due to uncertainty and overall burnout scores in general practice registrars. Additionally, the authors identified a positive correlation between the overall burnout score and the PRU subscale reluctance to disclose uncertainty to patients, which contradicts our results. We found a negative association between the PRU subscale and the OLBI scale exhaustion. As Cooke et al. (30) only used a single item to assess the burnout score, they did not differentiate core dimensions, which makes it difficult to compare differences. While our negative association appears counterintuitive initially, HCPs’ behavior might explain the association. Considering that patient demands are associated with burnout levels (18), greater reluctance to disclose uncertainty to patients could be interpreted as a coping behavior to avoid unpleasant situations with patients. It might lead to lower levels of exhaustion in the short term but can be adverse to the doctor-patient relationship in the long term (49). Communicating personal uncertainties to patients can be challenging for many reasons, including time pressure or fears of negative consequences when disclosing such uncertainties (50). By avoiding these difficult conversations, HCPs may find an effective way to deal with disclosing personal uncertainties. The potential pressure stemming from patient communication is emphasized by a longitudinal study conducted by Kapil et al. (51). The authors found a 2.7-fold increased risk for emotional exhaustion in HCPs who are in direct contact with patients compared to those HCPs who work in non-patient-facing roles.

### 4.3 Further influences of secondary factors

The weekly proportion of work with people with rare diseases was not associated with any dimension of uncertainty but seems to be a protective factor that is associated with lower disengagement. Due to the nature of the online survey, we can only speculate about the reasons for this association. In their scoping review, Llubes-Arrià et al. (52) described the diagnostic odyssey of many people with rare diseases and the moment of receiving a correct diagnosis as an important turning point. For patients, it means access to adequate treatment and specialists and the end of feelings of being misunderstood, fear, and helplessness. Our findings suggest that HCPs who regularly work with patients suffering from rare diseases may be more experienced or competent in providing adequate healthcare for this patient group. This positive interaction may contrast sharply with the experience of many patients, who often face challenges in being taken seriously regarding their conditions. Consequently, these experienced HCPs might be perceived as a vital source of support, fostering patient-physician relationships characterized by empathy and trust. A patient-physician relationship characterized by sympathy is associated with higher job satisfaction (53), which, in turn fosters greater work engagement (54). Building on this reasoning, factors such as patient satisfaction and empathy may influence the relationship between disengagement and the weekly proportion of work with people suffering from rare diseases. As previously mentioned, both constructs are related to burnout.

### 4.4 Gender effects

There were no direct effects of gender on burnout scores in our sample, but small indirect effects. The effects of gender on burnout are highly inconsistent (55). Similarly inconsistent are the effects of gender on uncertainty (22). We found female HCPs to report higher scores in three PRU subscales, but male HCPs scored higher in the subscale reluctance to disclose uncertainty to physicians.

## 5 Limitations

This study has several limitations. Since it was an online survey, we must assume a selection bias, making the generalizability of the results difficult. Due to our recruitment strategy, our analyses are based on a convenient sample. Second, the model provides no causal evidence about relationships between the parameters. The path directions are the only solution that fits our data and may be used to generate hypotheses to further examine the topic. The explorative nature of our study further underlines the necessity for additional research. Third, we only assessed the exposure to rare diseases with one self-report item. It might have been difficult for HCPs to adequately specify their weekly time with patients suffering from a rare disease. At the same time, our study has several strengths. This is the first study that examines uncertainty and burnout, considering the dimensions of both constructs. This allows us to analyze the associations in more detail. Other studies only use global scores for at least one of both constructs. The path analytical approach additionally allows us to generate ideas about the overall structure between the constructs’ dimensions instead of concentrating on single relationships. In addition, we managed to recruit various HCPs, and thus, the results were not limited to a certain specialization.

## 6 Conclusion

Our analyses highlights the importance of uncertainty when investigating burnout of HCPs. Anxiety due to uncertainty is especially associated with both core dimensions of burnout. This concludes the design of training for HCPs to learn how to cope with their uncertainties functionally to reduce their risk for burnout. One key component of such training should target relationship-focused strategies (56). Patients with rare diseases, in particular, are aware that HCPs may lack knowledge about their specific condition (49). While transparent communication is necessary to meet regulatory demands, it is challenging for many HCPs to disclose personal uncertainties (50). If successfully implemented, it can foster a stronger patient-physician relationship over time. This, in turn, may help reduce the risk of burnout among HCPs.

Additionally, it is essential to raise awareness and understanding of uncertainty and burnout in medical education. In their review, Ryan et al. (46) identified a culture of invulnerability among HCPs that hinders their ability to acknowledge personal weaknesses and seek support in addressing them. Normalizing issues associated with vulnerability, such as uncertainties and burnout, in medical education reaches as many (future) HCPs as possible and could encourage a culture that supports acknowledging one’s limits. This shift in collective attitude could serve as an important protective factor. Regularly working with patients suffering from rare diseases may also offer protective benefits, enhancing HCP engagement with their work. As our study design does not permit a deeper understanding of the associations involved, we can only speculate about influencing factors such as patient satisfaction and empathy. A thorough investigation of these aspects needs further exploration.

## Data Availability

The raw data supporting the conclusions of this article will be made available by the authors on reasonable request.

## Supplementary material

Supplementary Figure S1 Full-recursive model describing the relationships between exogenous variables (gender, age, proportion of work with rare diseases and COVID related stress), uncertainty (subscales of the Physicians’ Reaction to Uncertainty Scale), and burnout (subscales of the Oldenburg Burnout Inventory) in HCPs (*n*=128); eX represent error terms; path between proportion of work with rare diseases and COVID related stress was set to 1 to avoid underidentification.
