# Supplementary figures and images for: Exploring burnout and uncertainty in healthcare professionals: a path analysis within the context of rare diseases
Source: Front Public Health. 2025 Feb 27;13:1417771. doi: 10.3389/fpubh.2025.1417771 (PMC11903758; doi:10.3389/fpubh.2025.1417771)

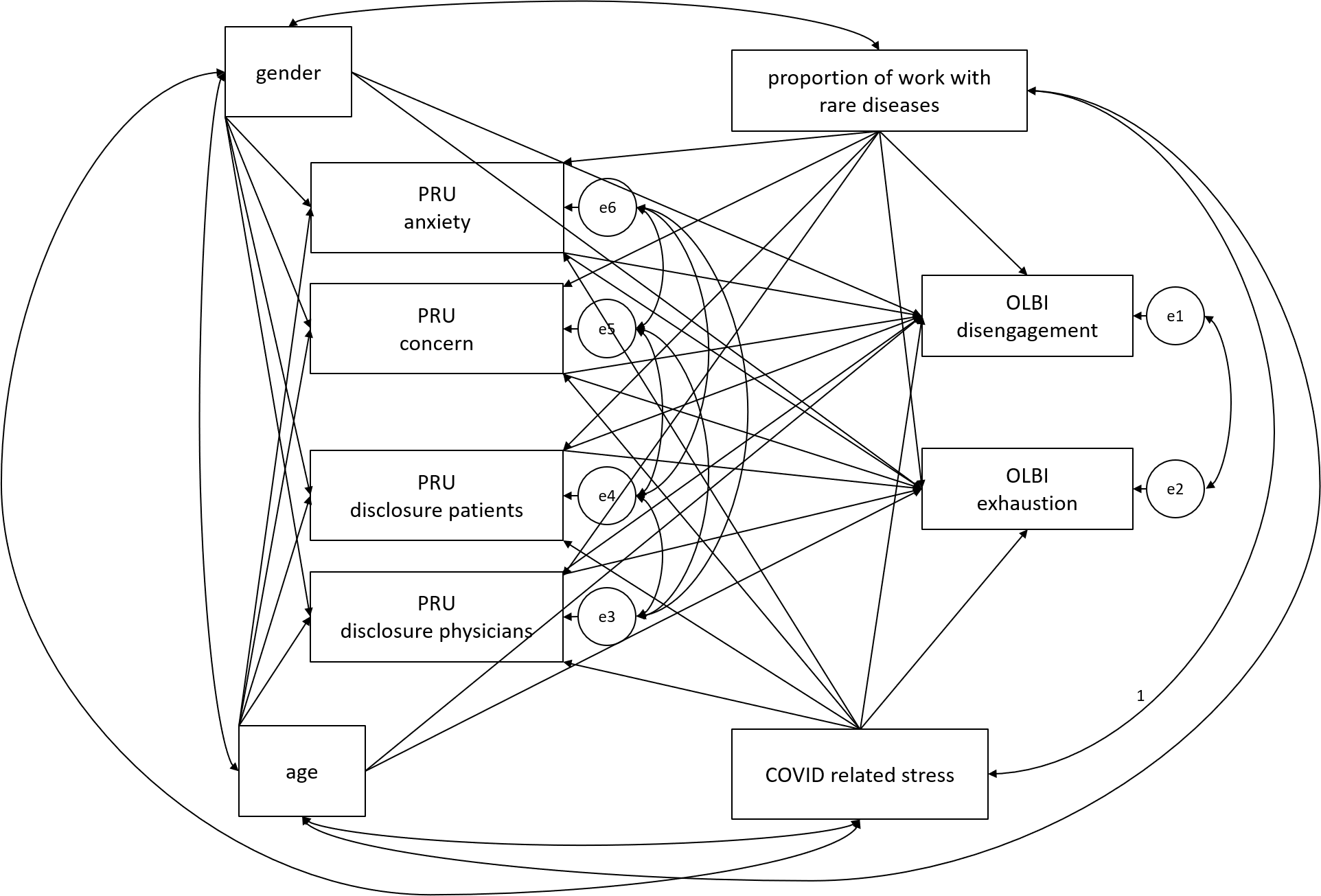

Supplement: Supplementary Figure S1 — Full-recursive model describing the relationships between exogenous variables (gender, age, proportion of work with rare diseases and COVID related stress), uncertainty (subscales of the Physicians’ Reaction to Uncertainty Scale), and burnout (subscales of the Oldenburg Burnout Inventory) in HCPs (n=128); eX represent error terms; path between proportion of work with rare diseases and COVID related stress was set to 1 to avoid underidentification. [file Image_1.tif]
