# Supplementary material for: Exploring burnout and uncertainty in healthcare professionals: a path analysis within the context of rare diseases
Source: Front Public Health. 2025 Feb 27;13:1417771. doi: 10.3389/fpubh.2025.1417771 (PMC11903758; doi:10.3389/fpubh.2025.1417771)
Supplement: Supplementary file 2 [file Table_1.docx]

**Additional File 2**

Comprehensive list of medical specialties

| Allergology |
| --- |
| Ophthalmology |
| (Neuro)surgery |
| Diabetology |
| Endocrinology |
| Gynaecology |
| Gastroenterology |
| Vascular medicine |
| Hematology |
| General and internal medicine |
| Dermatology |
| Ear, nose and throat medicine |
| Human genetics |
| Immunology |
| Cardiology |
| Pediatrics and adolescent medicine |
| Pulmonology |
| Neurology |
| Nephrology |
| Oncology |
| Orthopaedics |
| Pathology |
| Psychiatry |
| Psychosomatics |
| Radiology |
| Rheumatology |
| Pain therapy |
| Environmental medicine |
| Urology |
| Dentistry |
| Other medical specialty not mentioned |
